# High mRNA Expression of 24 Dehydrocholesterol Reductase (DHCR24) in the Treatment of Doxorubicin-Induced Heart Failure in Rats

**DOI:** 10.3390/ijms26010312

**Published:** 2025-01-01

**Authors:** Rui Zhang, Siyuan Peng, Xuejuan Zhang, Zhengwei Huang, Xin Pan

**Affiliations:** 1 School of Pharmaceutical Sciences, Sun Yat-sen University, Guangzhou 510275, China; zhangr358@mail.sysu.edu.cn (R.Z.); pengsy23@mail2.sysu.edu.cn (S.P.); 2 National-Local Joint Engineering Laboratory of Druggability and New Drugs Evaluation, Guangdong Provincial Key Laboratory of New Drug Design and Evaluation, Guangdong Province Engineering Laboratory for Druggability and New Drug Evaluation, School of Pharmaceutical Sciences, Sun Yat-sen University, Guangzhou 510006, China; 3 College of Pharmacy, Jinan University, Guangzhou 510632, China; zhanghongdou0223@126.com

**Keywords:** DHCR24, mRNA, LNP, heart failure

## Abstract

Objective: The objective of this study was to explore the possibility of treating heart failure in rats by delivering mRNA of 24-dehydrocholesterol reductase (DHCR24) into the body through lipid nanoparticles (LNPs). Methods: We established a heart failure rat model using doxorubicin. The experiment was divided into blank, model, mRNA stock solution cardiac injection, mRNA stock solution intravenous injection, LNP-mRNA stock solution cardiac injection, and LNP-mRNA stock solution intravenous injection groups. We directly injected DHCR24-mRNA or LNP-DHCR24-mRNA into the myocardium in three regions through an insulin needle passing through the intercostal space under the guidance of B-ultrasound. We recorded the mortality rate, body weight, 6-min walk test return times, and organ weight of rats after administration and detected the cardiac structure and function using B-ultrasound and transmission electron microscopy (TEM). Additionally, we tested for HE staining; PRDX2, Sirt3, and TRX1 protein expression; and IL-1 β, IL-10, VEGF, NT proBNP, and BNP cytokine concentrations. Results: Compared with the model group, the administration of DHCR24-mRNA significantly reduced mortality; decreased weight loss, the ratio of heart to tibia length, and spleen weight; and improved rat motility. The administration of DHCR24-mRNA can postpone the pathological morphological alterations of myocardial cells and reduce inflammatory infiltration. In terms of biochemistry, the administration of DHCR24-mRNA can increase the expression of the PRDX2, Sirt3, and TRX1 proteins; increase the concentrations of IL-10 and VEGF; and reduce the concentrations of IL-1β, NT proBNP, and BNP. The administration of DHCR24-mRNA can also delay the process of heart failure. The delivery and therapeutic effect of DHCR24-mRNA encapsulated in LNPs were better when compared to the other groups. Conclusions: DHCR24-mRNA encapsulated in LNPs can be effectively administered to rats with heart failure and exhibits some curative effects.

## 1. Introduction

Heart failure (HF) is a condition with a notably high incidence and mortality rate. Globally, 26 million individuals suffer from it [1,2,3]. Common treatment approaches typically involve the new quadruple therapy [4], such as the utilization of RAAS inhibitors and β-blockers [1,5,6]. Biomarkers B-type natriuretic peptide (BNP) and N-terminal pro-B-type natriuretic peptide (NT-proBNP), which are cardiac-protective hormones released by myocardial cells in response to stress, have been widely employed in the diagnosis and classification of heart failure [7]. Left ventricular ejection fraction (EF) is also a crucial parameter for the diagnosis and treatment of heart failure [8]. Research indicates that heart failure (HF) is closely related to inflammation. The plasma level of tumor necrosis factor alpha (TNF-α) is high in patients with HF, and some cytokines (such as IL-1β and IL-6) are also implicated in the process of heart failure. Currently, although there have been advancements in the treatment of heart failure (HF), the mortality rate and hospitalizations have not been substantially reduced [9,10], and the treatment methods for heart failure still represent an unmet clinical need [11,12]. 24-Dehydrocholesterol reductase (DHCR24) is a key regulatory factor involved in cholesterol synthesis and homeostasis [13] and is downregulated in the brain of Alzheimer’s disease (AD) models [14]. Furthermore, studies have demonstrated that DHCR24 is involved in amyloid-β deposition, tau protein hyperphosphorylation, autophagy [15], and apoptosis [16,17,18,19]. Apolipoprotein apoA-IV inhibits vascular inflammation in vivo and in vitro by suppressing the activation of NF-κB [20] in a DHCR24-dependent manner [21,22,23]. Moreover, increasing expression of DHCR24 can inhibit the generation of reactive oxygen species (ROS) [24], and DHCR24 possesses antioxidant and anti-apoptotic properties in acute lung injury (ALI) [21,23,25,26,27]. Ben J. Wu et al. discovered that high-density lipoproteins inhibit vascular endothelial inflammation by increasing DHCR24 reductase expression and inducing heme oxygenase-1 [28]. Fei Han et al. found that miR-124-targeting DHCR24 regulates oxidative stress and hypoxia-induced cardiomyocyte apoptosis and myocardial infarction [29]. PRDX2 is a member of the peroxidase family, and cholesterol is a product related to DHCR24 that is important for maintaining the integrity of cell membranes; maintaining a good membrane structure is crucial for the correct positioning and function of PRDX2. Sirt3 is a mitochondrial deacetylase involved in energy metabolism and antioxidant defense. It can sense and respond to changes in the cellular energy state and redox balance. Cholesterol is involved in the formation of lipid rafts in the cell membrane, which can affect mitochondria-associated signaling pathways. TRX1 plays a crucial role in the cellular redox regulatory network. Changes in membrane composition and function can affect the activity and localization of TRX1 [30,31]. DHCR24-mRNA treatment for heart failure may interact with PRDX2, Sirt3, and TRX1 through a variety of complex mechanisms, and it will be explored in the subsequent research. This study offers a preliminary exploration of the potential of DHCR24 as a therapeutic target for treating heart failure. Lipid nanoparticles (LNPs) are effective delivery vectors for mRNA, are widely utilized in vaccines [32] and can protect mRNA from enzymatic degradation with ubiquitous ribonucleases (RNases) [33,34]. LNP-mRNA anti-SARS-CoV-2 virus vaccines have attracted significant attention [29,32,33,35]. It is well known that mRNA therapy poses no risk of genomic integration and can be prepared rapidly [34,36,37].

Researchers and clinicians have been investigating mRNA therapeutics [38,39] for various diseases [32,40,41,42,43]. This study aims to explore the therapeutic effect of LNP-mRNA preparations of DHCR24 on rats with heart failure.

**Figure 1:**
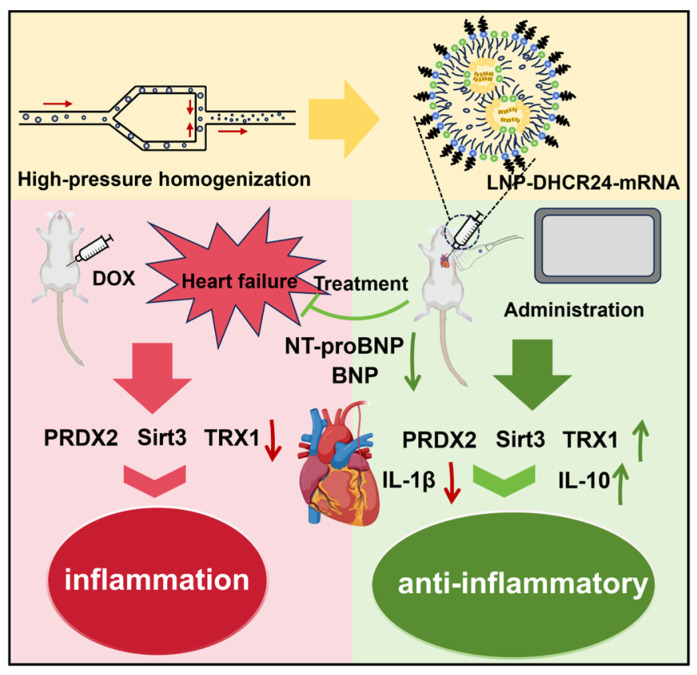
LNP-DHCR24-mRNA in the treatment of heart failure.

The main research contents are shown in Figure 1.

## 2. Results

### 2.1. Particle Size, Zeta Potential, and Electron Microscopy of LNP-mRNA of DHCR24

**Figure 2:**
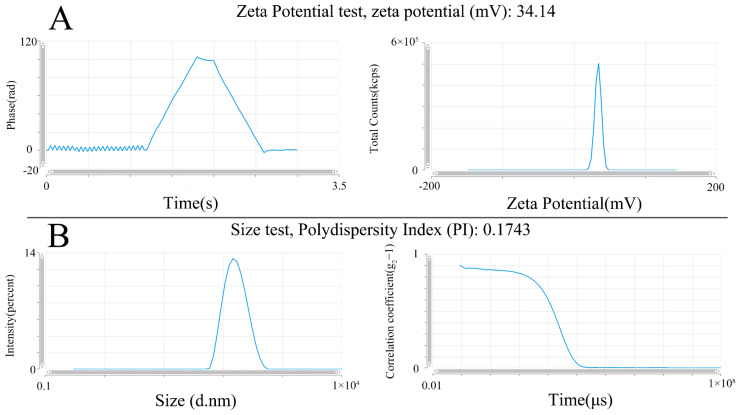
Characterization of the zeta potential and particle size distribution of LNP-DHCR24-mRNA: (**A**) zeta potential, which is used to observe the stability of LNPs; (**B**) particle size distribution and observed concentration of LNP size.

Zeta potential represents the stability of the sample, serving as a critical parameter. The greater the absolute value, the higher the stability of the formulation system. Generally, it needs to be greater than 30 mV. The zeta potential of LNP-DHCR24-mRNA in this experiment was 34.14 mV, indicating that this formulation was relatively stable. In the particle size test, the smaller the PI value, the narrower the particle size distribution. Generally, a PI value less than 0.3 indicates a relatively concentrated particle size distribution, and a value between 0.3 and 0.7 is relatively poor. The PI value of 0.1743 for the formulation in this study indicated a favorable particle size distribution (Figure 2).

**Figure 3:**
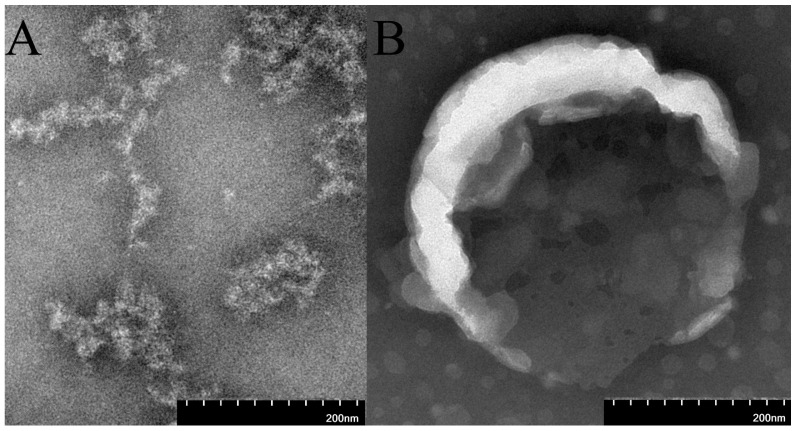
Transmission electron microscope characterization of DHCR24-mRNA (**A**) and LNP-DHCR24-mRNA (**B**).

Under the TEM, DHCR24-mRNA appeared as a linear structure, accompanied by the phenomena of winding or aggregation. However, its overall morphology was relatively simple, and there was no obvious regular spherical or vesicular structure. LNP-DHCR24-mRNA presented a vesicular structure (Figure 3).

### 2.2. The mRNA and Protein Levels of DHCR24 in the H9C2 Rat Cardiomyocytes

**Figure 4:**
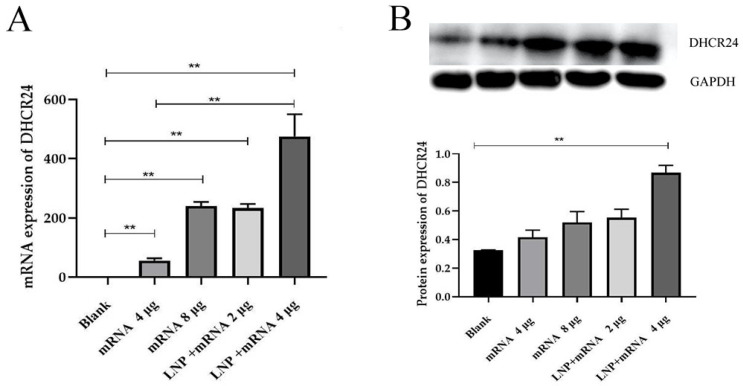
The mRNA and protein levels of DHCR24 in each administration group of H9C2 rat cardiomyocytes: (**A**) qPCR results for DHCR24 mRNA; (**B**) protein results of DHCR24-mRNA. Data are expressed as the mean ± SD. ** *p* < 0.01 compared to the blank group.

Cardiomyocytes have the potential to phagocytose certain substances, but whether they specifically phagocytose LNP-DHCR24-mRNA and take it into the cells depends on several factors. LNPs are designed to protect and deliver nucleic acids, such as mRNA. Their size, surface charge, and composition can influence their interaction with cells. Cardiomyocytes possess various endocytic pathways that could potentially be involved in the uptake of the LNP–mRNA complex, including clathrin-mediated endocytosis, caveolae-mediated endocytosis, and macropinocytosis. The specific uptake mechanism may depend on the properties of the LNPs and the cellular environment [32]. Before conducting the animal experiments, we first verified the cell delivery and expression of DHCR24-mRNA using H9C2 rat cardiomyocytes. The results show that LNPs can significantly (*p* < 0.01) enhance the cell delivery efficiency of DHCR24-mRNA (Figure 4).

### 2.3. In Vivo Evaluation of Therapeutic Efficacy in a Rat Myocardial Injury Model

**Figure 5:**
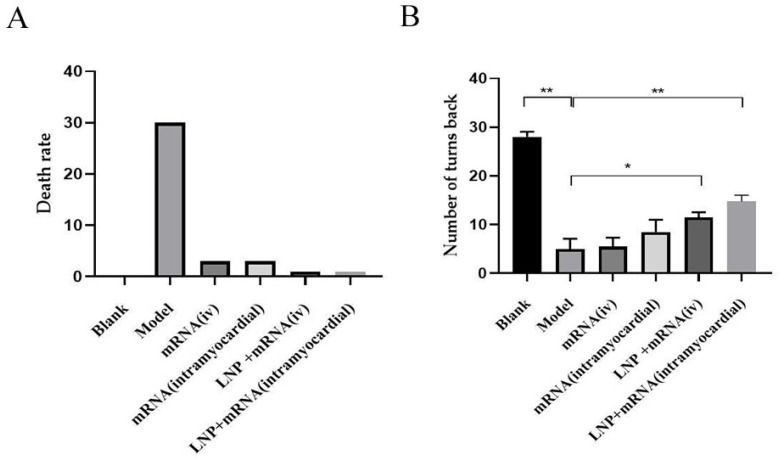
Comparison of the mortality rate and 6-min walk test results for each treatment group with those of the model group: (**A**) mortality rate of each group; (**B**) number of turns for each group. Data are expressed as the mean ± SD. * *p* < 0.05 and ** *p* < 0.01 compared to the model group.

Direct delivery of a DHCR24-mRNA stock solution or LNP formulation of DHCR24-mRNA can significantly reduce the mortality rate of the rats with heart failure compared to the model group. It can be seen from the 6-min walk experiment that the myocardial injection of DHCR24-mRNA stock solution delayed the decrease in exercise capacity of rats with heart failure compared to the model group, although the difference was not significant. The administration of LNP-DHCR24-mRNA significantly enhanced the exercise capacity of the rats with heart failure, especially in the myocardial injection group (Figure 5).

**Figure 6:**
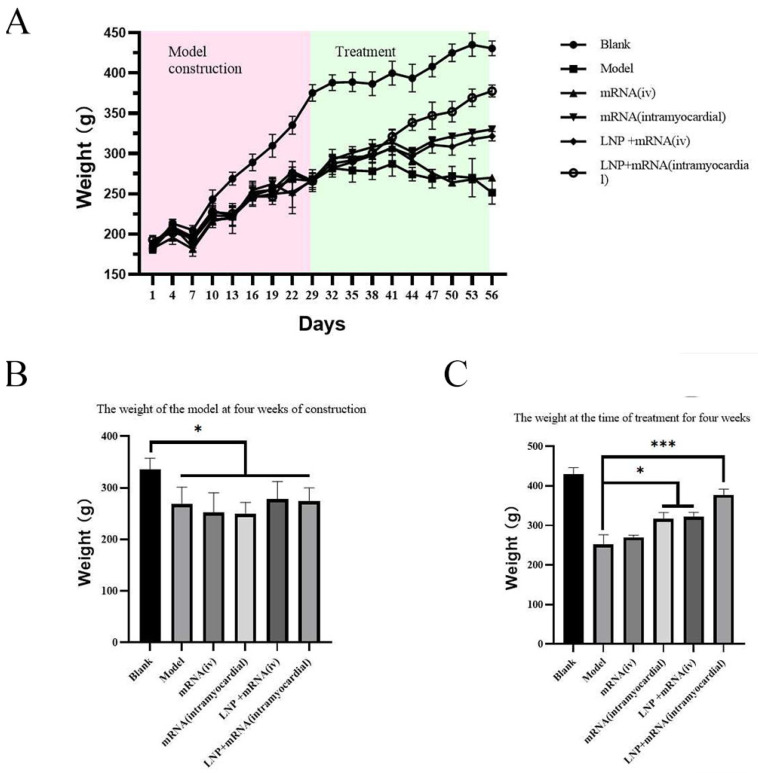
Body weight of each group of rats before and after modeling, as well as before and after administration: (**A**) weight during the modeling and treatment periods; (**B**) weight of the model at four weeks of construction; (**C**) weight at the time of treatment for four weeks. Data are expressed as the mean ± SD. * *p* < 0.05 and *** *p* < 0.005compared to the blank or model group.

**Figure 7:**
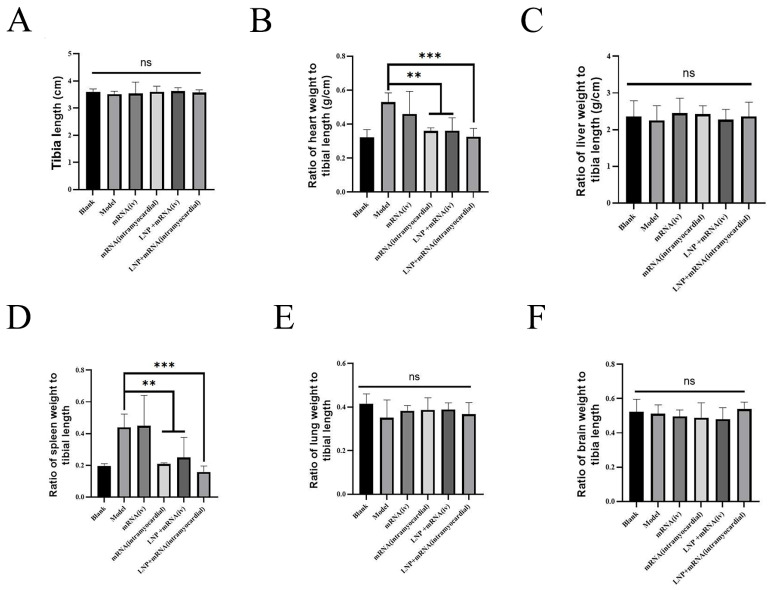
Tibia length of each group of rats and the ratio of each tissue to tibia length: (**A**) tibia length; (**B**) ratio of heart weight to tibia length; (**C**) ratio of liver weight to tibia length; (**D**) ratio of spleen weight to tibia length; (**E**) ratio of lung weight to tibia length; (**F**) ratio of brain weight to tibia length. Data are expressed as the mean ± SD. ns, not significant; ** *p* < 0.01 and *** *p* < 0.001 compared to the model group.

Twenty-nine days after modeling with doxorubicin, the body weight of model rats decreased significantly compared with that of the control group. After the administration of DHCR24-mRNA, compared with the model group, the DHCR24-mRNA stock solution myocardial injection group and the LNP-DHCR24-mRNA intravenous injection group presented a significant increase in body weight, and the effect on the LNP-DHCR24-mRNA myocardial injection group was more pronounced (Figure 6). Moreover, compared with that of the DHCR24-mRNA myocardial injection group, the body weight of the LNP-DHCR24-mRNA myocardial injection group increased significantly. There were no significant differences in tibia length, liver, lung, or brain among each group of rats. Compared with the model group, the DHCR24-mRNA stock solution myocardial injection group and the LNP-DHCR24-mRNA intravenous injection group had significantly reduced heart/tibia and spleen/tibia ratios, and the effect on the LNP-DHCR24-mRNA myocardial injection group was more obvious. The heart/tibia and spleen/tibia ratios in the LNP-DHCR24-mRNA myocardial injection group showed a downward trend compared with those in the DHCR24-mRNA stock solution myocardial injection group, but there was no significant difference (Figure 7).

### 2.4. Inflammatory Infiltration Reduced by LNP-DHCR24-mRNA

**Figure 8:**
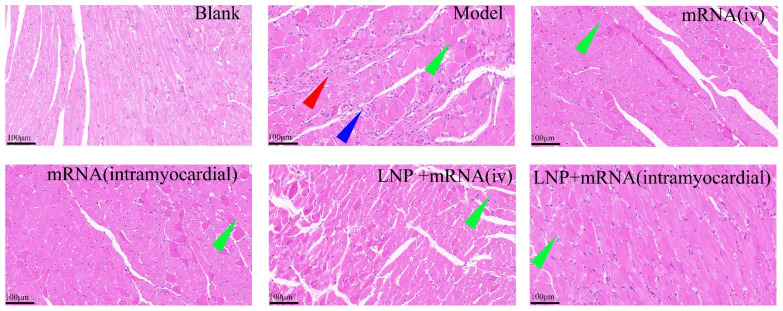
Histopathological changes in heart tissue in heart failure rat model using HE staining. The red arrows mark the areas of myocardial necrosis, the green arrows mark the areas of myocardial hypertrophy, and the blue arrows mark the areas of inflammatory cell infiltration.

In the control group of rats with normal heart function, the cardiac tissue structure appeared clear, and the cellular morphology was normal. Conversely, in the model group, the myocardial cells exhibited hypertrophy, with evident fractures, necrosis, degeneration, and inflammatory infiltration; compared with the model group, the DHCR24-mRNA intravenous and stock solution myocardial injection groups and the LNP-DHCR24-mRNA intravenous and myocardial injection groups all showed different degrees of recovery in myocardial cell morphology, hypertrophy, and inflammatory infiltration, among which the effect of the LNP-DHCR24-mRNA myocardial injection group was more remarkable (Figure 8).

**Figure 9:**
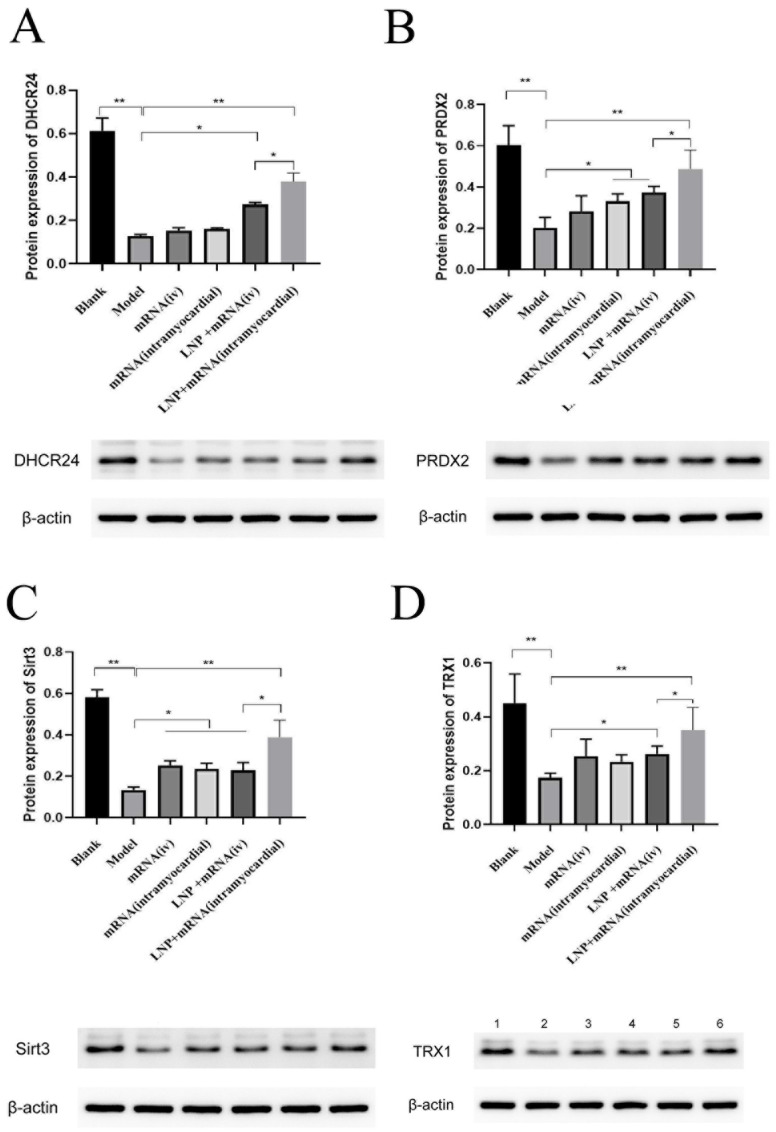
Expression of DHCR24 in cardiac tissue and the effects of administration of DHCR24-mRNA on the protein expression levels of PRDX2, Sirt3, and TRX1 in the tissues of rats with heart failure: (**A**) protein expression of DHCR24 within cardiac tissue; (**B**) protein expression of PRDX2 within cardiac tissue; (**C**) protein expression of Sirt3 within cardiac tissue; (**D**) protein expression of TRX1 within cardiac tissue. Data are expressed as the mean ± SD. * *p* < 0.05 and ** *p* < 0.01 compared to the blank or model group.

Upon the administration of DHCR24-mRNA, it was observed that, in comparison to the model group, both the intravenous and myocardial injection groups receiving the DHCR24-mRNA stock solution, as well as the intravenous and myocardial injection groups treated with LNP-DHCR24-mRNA, exhibited varying levels of enhanced DHCR24 expression. Among them, the use of LNP encapsulation enhanced the delivery efficiency. The protein expression levels of PRDX2, Sirt3, and TRX1 in each DHCR24-mRNA administration group all increased to different extents. Moreover, compared with the DHCR24-mRNA myocardial injection group, the LNP-DHCR24-mRNA myocardial injection group had a significant difference in the extent of elevation of three proteins (Figure 9).

**Figure 10:**
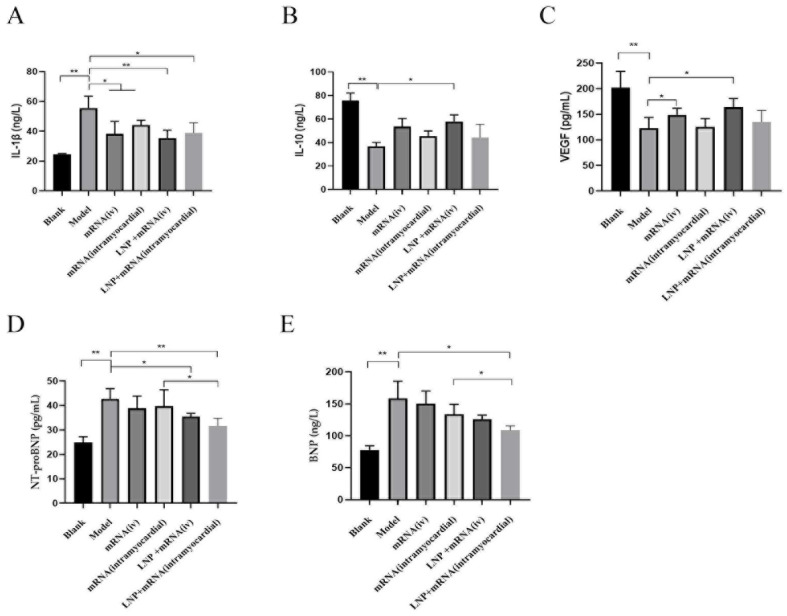
Effects of administration of DHCR24-mRNA on the expression of IL-1β, IL-10, VEGF, NT-proBNP, and BNP in the blood of rats with heart failure: (**A**) expression of IL-1β in the blood of rats from each group; (**B**) expression of IL-10 in the blood of rats from each group; (**C**) expression of VEGF in the blood of rats from each group; (**D**) expression of NT-proBNP in the blood of rats from each group; (**E**) expression of BNP in the blood of rats from each group. Data are expressed as the mean ± SD. * *p* < 0.05 and ** *p* < 0.01 compared to the blank or model group.

After the administration of DHCR24-mRNA, compared with the model group, the DHCR24-mRNA stock solution intravenous and myocardial injection groups and the LNP-DHCR24-mRNA intravenous and myocardial injection groups all had different degrees of reduction in the expression levels of IL-1β, NT-proBNP, and BNP, while the levels of IL-10 and VEGF all increased to different degrees. The effect of using the LNP encapsulation method was more conspicuous. In terms of inflammatory factors, there was no significant difference between the LNP-DHCR24-mRNA and DHCR24-mRNA myocardial injection groups. However, in terms of NT–proBNP and BNP detection, the results of the LNP-DHCR24-mRNA myocardial injection group showed a significant decrease compared with those of the DHCR24-mRNA myocardial injection group (Figure 10).

### 2.5. The Pathological Morphological Alterations of Myocardial Cells Postponed by LNP-DHCR24-mRNA

**Figure 11:**
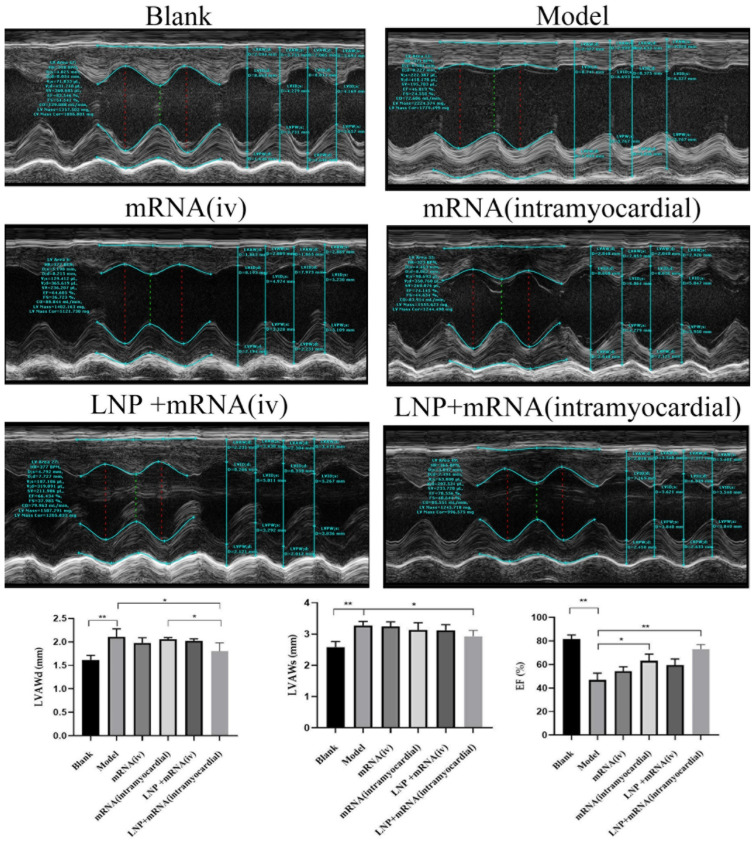
The B-ultrasound situation of each group of rats on the 27th day after administration of DHCR24-mRNA. The red dotted line and the green dotted line represent the distances of the left ventricular cavity during diastole and systole respectively. Data are expressed as the mean ± SD. * *p* < 0.05 and ** *p* < 0.01 compared to the blank or model group.

On the 27th day post-administration of DHCR24-mRNA, in comparison with the model group, only the LNP-DHCR24-mRNA myocardial injection group had a significant reduction in the diastolic thickness of the anterior wall of the left ventricle (LVAWd) and the systolic thickness of the anterior wall of the left ventricle (LVAWs); the ejection fraction (EF) increased for each administration group, among which the increase for the LNP-DHCR24-mRNA myocardial injection group was more evident. There was no significant difference between LNP-DHCR24-mRNA and DHCR24-mRNA myocardial injection groups in the detection of LVAWs and EF%. However, in the detection of LVAWd, the results of the LNP-DHCR24-mRNA myocardial injection group showed a significant decrease compared with those of the DHCR24-mRNA myocardial injection group (Figure 11).

As shown in Figure 11, the heart tissues of the rats in each group were evaluated using the transmission electron microscopy (TEM).

Blank: The tissue structure was essentially normal. The myocardial fibers observed within the field of vision were neatly and tightly arranged. The mitochondria were abundant and evenly distributed, exhibiting a distinct double-layer membrane structure. Lamellar cristae were observable, and the cristae protrusions were roughly arranged in parallel lamellar patterns. The blue arrow indicates the I band Z line, the green arrow indicates the A band M line, and the black arrow indicates the myofilament. The myofilaments were neatly and tightly arrayed to form sarcomeres, and no fractures were detected.

Model: The tissue structure was severely abnormal. The myocardial fibers within the field of vision were disorderly arranged and locally dissolved and fractured, as indicated by the black arrow. Some mitochondria were swollen, and the electron density was reduced, as indicated by the red arrow. Some mitochondrial membranes were ruptured, as indicated by the orange arrow. A considerable number of autophagosomes can also be seen, as indicated by the yellow arrow. The blue arrow indicates the I band Z line, and the green arrow indicates the A band M line.

mRNA (iv): The tissue structure was moderately abnormal. The myocardial fibers within the field of vision were loosely arranged and locally dissolved and fractured, as indicated by the black arrow. A small quantity of mitochondria were swollen, and the electron density was reduced, as indicated by the red arrow. A small number of autophagosomes can also be seen, as indicated by the yellow arrow. The blue arrow indicates the I band Z line, and the green arrow indicates the A band M line.

mRNA (intramyocardial): The tissue structure was mildly abnormal. The myocardial fibers within the field of vision were relatively tightly arranged and locally dissolved and fractured, as indicated by the black arrow. The mitochondria were abundant and evenly distributed, with a clear double-layer membrane structure. Lamellar cristae were visible, and the cristae protrusions were approximately arranged in parallel lamellar shapes. The blue arrow indicates the I band Z line, and the green arrow indicates the A band M line.

LNP + mRNA (iv): The tissue structure was mildly abnormal. The myocardial fibers within the field of vision were relatively tightly arranged and locally dissolved and fractured, as indicated by the black arrow. The mitochondria were abundant and evenly distributed, with a clear double-layer membrane structure. Lamellar cristae were observable, and the cristae protrusions were approximately arranged in parallel lamellar shapes. The blue arrow indicates the I band Z line, and the green arrow indicates the A band M line.

**Figure 12:**
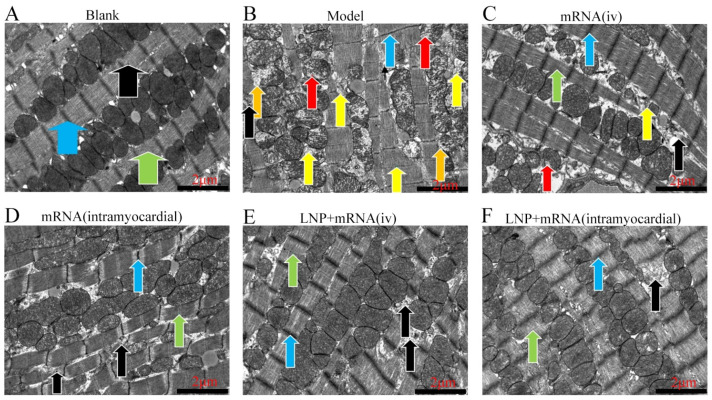
TEM image of heart tissues of each group of rats: (**A**) results of the blank group; (**B**) results of the model group; (**C**) results of the mRNA stock solution intravenous injection group; (**D**) results of the mRNA stock solution cardiac injection group; (**E**) results of the LNP-mRNA stock solution intravenous injection group; (**F**) results of the LNP-mRNA stock solution cardiac injection group. The blue arrow indicates the Z-line of the I-band of myocardial cells; the green arrow represents the M-line of the A-band; the black arrow shows the myofilament; the red arrow stands for the swelling of some mitochondria; the orange arrow represents the rupture of the membranes of some mitochondria; the yellow arrow symbolizes the autophagosome.

LNP + mRNA (intramyocardial): The tissue structure was slightly abnormal. The myocardial fibers within the field of vision were relatively tightly arranged and locally dissolved, as indicated by the black arrow. The mitochondria were abundant and evenly distributed, with a clear double-layer membrane structure. Lamellar cristae were visible, with their protrusions arranged in approximately parallel lamellar patterns. The blue arrow indicates the I band Z line, the green arrow indicates the A band M line, and no fractures in the sarcomeres were found (Figure 12).

## 3. Discussion

LNPs have become a commonly used carrier for nucleic acid drug delivery [40] and have gradually entered clinical application. In this study, the LNP-DHCR24-mRNA formulation exhibited a zeta potential of 34.14 mV and a PI value of 0.1743 (Figure 2). The TEM images (Figure 3) showed that DHCR24-mRNA had a linear structure and was accompanied by winding or aggregation, while LNP-DHCR24-mRNA had a vesicular structure. These findings indicated that the LNP-DHCR24-mRNA had a narrow particle size distribution. Moreover, it also demonstrated high efficiency in cell and tissue delivery (Figure 4). A high delivery rate will improve its efficacy, enabling a reduction in the dosage and thereby reducing adverse reactions [33].

Symptoms of patients with heart failure include coughing and wheezing, as well as a decline in exercise capacity. Exercise capacity is directly related to the quality of life of patients [13]. In this study, through the administration of DHCR24-mRNA, in comparison with the model group, it was possible to postpone the decline in exercise capacity (Figure 5). Moreover, the myocardial injection group with LNP showed the best effect. The non-LNP myocardial injection group and the LNP intravenous injection group had comparable effects, but without significant differences. The non-LNP intravenous injection group did not exhibit a therapeutic effect compared to the model group, which is related to the relatively rapid metabolism of mRNA in vivo after intravenous injection. In addition, the body weight of the rats with heart failure was restored, and the mortality rate was significantly reduced. The body weight of the LNP-DHCR24-mRNA myocardial injection group increased significantly compared to that of the other groups. This might be associated with the improvement in cardiac function, which subsequently enhanced the appetite of the rats with heart failure (Figure 6).

The size of the heart was calculated in relation to the length of the tibia in order to eliminate the interference of body size differences in the assessment of heart size. In heart failure, the decreased pumping ability of the heart leads to congestion in the hepatic veins, which may cause damage to liver cells. Due to the backflow of blood, the liver may become enlarged. The spleen is involved in the immune system and its functions, and it becomes enlarged when there are immune problems such as inflammation. In heart failure, blood flows back to the pulmonary circulation, resulting in pulmonary congestion. This may lead to shortness of breath, coughing (especially a wet cough caused by fluid in the lungs), and a decline in lung function. In severe heart failure, the reduction in cardiac output leads to a decrease in the blood supply to the brain, which may cause cerebral hypoxia (lack of oxygen in the brain). The hearts of patients with heart failure usually become hypertrophic. After the administration of DHCR24 mRNA, the heart/tibia ratio was reduced. During immune responses such as inflammation, the weight of the spleen increased accordingly. After the administration of DHCR24-mRNA, the spleen weight was restored, indicating that DHCR24-mRNA inhibited the inflammatory response, which is consistent with the results of HE and ELISA. The heart/tibia and spleen/tibia ratios in the LNP-DHCR24-mRNA intravenous injection group, the LNP-DHCR24-mRNA myocardial injection group, and the DHCR24-mRNA myocardial injection group all showed a downward trend. However, there was no significant difference between the LNP-DHCR24-mRNA and DHCR24-mRNA myocardial injection groups (Figure 7). From this result, it can be seen that myocardial injection can substitute for part of the delivery function of LNP.

Lisa Hasselbach et al. studied the relationship between heart failure and inflammation [11]. The results of this article are similar. Studies have found that SIRT3, Trx1, and PRDX2 are beneficial for coping with oxidative stress during the aging process [44,45,46]. In this study, after DHCR24-mRNA was administered, the expression of these three proteins increased, which is similar to the above results. Compared with in the DHCR24-mRNA myocardial injection group, there were significant differences in the elevation levels of the three proteins in the LNP-DHCR24-mRNA myocardial injection group (Figure 9). This might be due to the fact that after DHCR24-mRNA is encapsulated by LNP, the difficulty of its degradation increases, and accordingly, the anti-inflammatory effect is enhanced. IL-1β is related to inflammation and autoimmunity. IL-10 can inhibit the secretion of TNF-α, IL-1β, IL-6, IL-8, G-CSF, and GM-CSF. A lack of IL-10 leads to continuous immune activation, thereby triggering chronic inflammatory bowel diseases (such as Crohn’s disease) [47]. Vascular endothelial growth factor (VEGF) has the function of promoting angiogenesis [48]. Both NT-proBNP and BNP are markers of heart failure [49]. The findings of this study indicated that there was a partial enhancement in the levels of these cytokines following the delivery of DHCR24 mRNA, particularly when LNPs were utilized. Moreover, this is consistent with the spleen weight and HE staining results. At the molecular biology level, the possible mechanism of DHCR24-mRNA in treating heart failure is related to its anti-inflammatory and angiogenesis-promoting activities. There was no significant difference in terms of inflammatory factors in the DHCR24-mRNA myocardial injection group with or without LNPs. However, in the detection of NT-proBNP and BNP, the group with LNPs showed a better effect (Figure 10). Because NT-proBNP and BNP are important biomarkers of heart failure, the results suggest that adding LNP might be a preferable choice.

Cardiac B-ultrasound is commonly utilized to detect the structure and function of the heart. The indicators explored in this study, such as the diastolic thickness of the anterior wall of the left ventricle (LVAWd), the systolic thickness of the anterior wall of the left ventricle (LVAWs), and the ejection fraction (EF), showed that DHCR24-mRNA can delay the process of heart failure. For DHCR24-mRNA myocardial injection, whether with or without LNPs, there was no significant difference in terms of LVAWs and EF%. However, adding LNPs could significantly reduce the LVAWd (Figure 11). This might be because the thickness of the cardiac wall at the end of diastole was thinned, making it easier to show the difference.

The transmission electron microscopy results showed that compared with the blank group, in the model group, myocardial fibers were in a disorderly arrangement and locally dissolved and broken. Some mitochondria were swollen, the mitochondrial membrane was ruptured, and there were more autophagosomes. Compared with the model group, the mRNA stock solution intravenous injection group showed loosely arranged myocardial fibers, reduced mitochondrial damage, and fewer autophagosomes. Compared with the model group, the mRNA stock solution cardiac injection group and the LNP-mRNA stock solution intravenous injection group showed no abnormalities in mitochondria. In the LNP-mRNA stock solution cardiac injection group, myocardial fibers were locally dissolved but not broken, which was closest to the blank group. The above results indicate that administering DHCR24-mRNA can protect and repair cardiomyocytes at the cellular structure level. This is similar to the research methods of Peipei Zhang et al. [50].

The potential advantages of LNP-DHCR24-mRNA through myocardial injection are as follows: (1) Gene-specific regulation: In cases of heart failure, there may be defects or dysregulation in DHCR24 expression, and this could be a more direct way to address the root cause. (2) Cell-specific targeting: Lipid nanoparticles (LNPs) can be designed to target specific cell types in the heart, ensuring that the therapeutic effect is concentrated on the cells that are crucial for cardiac function. LNP-DHCR24-mRNA myocardial injection can achieve a relatively high local concentration of mRNA in the heart tissue, avoid the first-pass effect, and reduce drug side effects. (3) Possible new mechanism of action: By restoring normal DHCR24 function, it may help maintain the integrity of the cardiomyocyte cell membrane, which is vital for normal cell signaling and function in the context of heart failure.

Although this experiment investigated the possibility of treating heart failure through the high expression of LNP-mRNA in DHCR24, there are numerous design and research deficiencies. For instance, the effects of different LNP ratios on mRNA delivery and the expression of DHCR24 were not explored. Additionally, the impact of DHCR24-mRNA on cholesterol expression was not examined, and an orthogonal comparative design was not considered. Moreover, the DHCR24-mRNA mechanism of action has not been thoroughly investigated, and the characteristics measured for 60 rats in two groups were not recorded, namely the model construction and treatment groups. In addition, when contrasted with intravenous injections, medications administered directly into the myocardium predominantly remain localized to the area of injection, exhibiting a limited dispersion radius. This targeted delivery approach ensures a high concentration of drugs at the site of myocardial lesions, which can be advantageous for treating localized myocardial injuries. However, myocardial injections are not without their risks; they may induce arrhythmias due to the potential stimulation of myocardial cells’ electrical activity during the injection process. Moreover, there are very few direct reports on the relationship between DHCR24 and heart failure. Initially, we only intended to preliminarily explore its drug efficacy, so the design of positive control drugs was overlooked. Furthermore, examining the alterations in cholesterol levels within each group is of paramount importance. This approach can yield more direct evidence supporting the functional outcomes of DHCR24 modulation and offer a more comprehensive understanding of the overall impact on lipid metabolism in the context of doxorubicin-induced heart failure. We will continue to address these shortcomings in future research.

In addition, clinically, patients have a higher acceptance of intravenous injection. We mainly choose myocardial injection because it can reduce the drug administration dosage. Reducing the dosage can, in turn, minimize potential side effects and drug costs (currently, the cost of nucleic acid drugs is relatively high). However, with the increasing number of nucleic acid drug production factories, their cost may also decline. In the future, after sufficient toxicological studies are conducted, we also intend to use intravenous injection to improve patient compliance.

## 4. Materials and Methods

### 4.1. Materials

Animals: Sixty male SD rats (male rats typically display more consistent responses in specific physiological and pathological processes, enhancing the reproducibility of experimental outcomes), weighing within the range of 220 to 250 g, were procured from Jinfeng Laboratory Animal Co., Ltd., in Jinan, China. All experimental studies were approved by the Animal Ethics Committee of the Heze Institute for Food and Drug Control. The Heze Testing Research Institute for Food and Drug Control boasts a GLP-compliant experimental animal facility and has collaborated with our team in conducting this research. Consequently, all animal feeding and sampling procedures were performed within the Institute’s premises in Heze (approval ID: 20233481346; date: 27 April 2023).

The mRNA preparation of DHCR24 was self-generated using in vitro synthesis. Pentobarbital sodium was provided by Sinopharm Chemical Reagent Company, in Guangzhou, China. Doxorubicin was sourced from Aladdin. LNP materials were provided, and their preparation was accomplished by Shanghai University. The production process of the LNP-DHCR24-mRNA drug can be roughly divided into steps such as plasmid production, plasmid linearization, in vitro transcription synthesis reaction, mRNA purification, and encapsulation. The plasmid is the raw material for mRNA drug production, carrying the genetic information-encoding DHCR24. It was transferred into Escherichia coli to construct a strain library, and the passage stability was studied. Subsequently, the strain library was amplified, the plasmid was extracted, and a linearized plasmid for in vitro transcription (IVT) use was prepared. With the help of IVT technology, DHCR24-mRNA was transcribed using the linearized plasmid as a template, mainly including process steps such as transcribing the linearized plasmid DNA into mRNA, separation, and purification. After that, 300 μL of 2.5 M CaCl_2_ and 60 μg of DHCR24-mRNA were dispersed in 20 mL of cyclohexane/Igepal CO-520 (69:31, V/V) solution to form the calcium phase. Then, 300 μL of 12.5 mM Na₂HPO₄ was added to 20 mL of cyclohexane/Igepal CO-520 (69/31, V/V) solution to form the phosphate phase. Next, 100 μL of DOPA (20 mg/mL) in chloroform was added to the phosphate phase. After the two phases were mixed uniformly, ethanol was added, followed by centrifugation at 12,500× *g* for 30 min to remove the cyclohexane/Igepal CO-520. After three washes with ethanol, the CaP core pellets were dissolved in 2 mL of chloroform and stored in glass vials until use. Next, 500 μL of the abovementioned CaP cores and the lipid constituents (DOTAP, DOPE, cholesterol, and DSPE—PEG2000 at a molar ratio of 0.8:1:0.5:0.09) was dissolved in chloroform. The chloroform was removed via rotary evaporation under vacuum. The suspension was intermittently sonicated and then extruded through a polycarbonate membrane to obtain LNP-DHCR24-mRNA. Phosphotungstic acid was sourced from Rhawn. H9C2 rat cardiomyocytes were provided by Shanghai Hongshun Biotechnology Co., Ltd. (Shanghai, China). DHCR24 antibody was sourced from Affinity, San Francisco, CA, USA. DHCR24 primer design was provided by Nanjing Kangxuhe Biotechnology Co., Ltd. (Nanjing, China). An SDS-PAGE gel preparation kit was provided by New Cellmax. In total, 0.45 μm of PVDF membrane was used. A BCA protein concentration determination kit was provided by Biosharp (Hefei, China). A protein Marker was sourced from Solarbio (Beijing, China). An Animal Total RNA Isolation Kit was bought from Sevier. A 2× Fast SYBR Green qPCR Master Mix was purchased from Sevier (Suresnes, France). A SweScript RT II First Strand cDNA Synthesis Kit was purchased from Sevier. ELISA kits for rat interleukin 10 (IL-10), 1β (IL-1β), rat vascular endothelial growth factor (VEGF), rat N-terminal brain natriuretic peptide (NT-proBNP), and rat brain natriuretic peptide/brain natriuretic peptide (BNP) were all purchased from Enzyme Immunoassay Biological Company in Wuhan, China.

### 4.2. Instruments

An LNP microfluidic preparation instrument and a microfluidic LNP preparation instrument homogenizer were purchased from Nazhida (Shanghai) Nanotechnology Co., Ltd. (Shanghai, China). A transmission electron microscope was purchased from Thermo Fisher (Waltham, MA, USA). A zeta potential nanometer particle size analyzer was purchased from Malvern (Malvern, UK). A microplate reader was purchased from Tecan (Männedorf, Switzerland). A transmembrane instrument was purchased from Liuyi Company (Beijing, China). A fully automatic chemiluminescence image analysis system was purchased from Shanghai Tianneng (Shanghai, China). A low-temperature high-speed centrifuge was purchased from Xiangyi (Shanghai, China). An ultraviolet spectrophotometer was purchased from Implen (Munich, Germany). A fluorescence quantitative PCR instrument was purchased from BIO-RAD (Hercules, CA, USA).

### 4.3. Methods

#### 4.3.1. Grouping, Modeling, and Administration

This research sought to investigate the therapeutic efficacy disparities between intravenous and myocardial injections. The experiment was divided into 6 groups: the blank group, the model group, the mRNA stock solution cardiac injection group, the mRNA stock solution intravenous injection group, the LNP-mRNA stock solution cardiac injection group, and the LNP-mRNA stock solution intravenous injection group. There were 10 rats in each group. For model establishment, adriamycin (ADR) was injected intraperitoneally at a dose of 2.5 mg/kg once every 4 days for a total of 29 days. The rats in the blank group were administered normal saline. The concentration of LNP-mRNA and mRNA stock solution was 47 μg/mL. For both the myocardium and the vein, 60 μg doses were administered using insulin needles. The administration was given once, and observations were carried out for 27 days. During this period, the body weight was measured, and the mortality rate was recorded. After 27 days, the blood and cardiac tissue were collected, and the tibia length was measured. We performed direct myocardial injection under ultrasound guidance. We carefully inserted the insulin needle through the intercostal space and injected the drug into the myocardial area in three injection points with the help of B-ultrasound visualization. Doxorubicin possesses potent toxic side effects, including but not limited to arrhythmia, intestinal distension, gastric hemorrhage, and ascites. The challenges encountered during experimental procedures also played a significant role in the elevated mortality rate among rats. For instance, an improper injection technique when administering doxorubicin, which results in organ damage, could further contribute to rat fatalities. We performed necropsies on deceased rats to ascertain the cause of death. When tallying the number of rat fatalities, we exclusively accounted for those that succumbed due to doxorubicin. Furthermore, we standardized other variables to remain consistent, such as the feeding environment, post-administration care, feed, and drinking water, with the only variation being in the medication administration.

#### 4.3.2. Zeta Potential

Clean sample containers that were compatible with the zeta potentiometer were prepared. An appropriate amount of the sample was taken. The sample that had no visible contaminants or aggregates was ensured. The zeta potentiometer software which model is ZS Xplorer was opened. The sample container was placed in the sample holder of the zeta potentiometer. Measurement parameters, such as temperature (25 °C), number of measurements (automatic), and measurement duration (3 times), were set. The measurement was started, and the zeta potential and particle size data of the sample were collected by the instrument. The data were analyzed and recorded.

#### 4.3.3. Transmission Electron Microscope

The acceleration voltage was 120 kilovolts. The magnification was 100,000 times. The resolution was 0.2 nm. The preparation structure of LNP-mRNA encapsulated was observed with DHCR24 using LNPs (LNP-DHCR24-mRNA), mRNA of DHCR24 without LNPs (DHCR24-mRNA) and the ultrastructure of cardiomyocytes and heart tissue. Experimental steps of the negative staining technique: a drop of 10 μL of DHCR24-mRNA and LNP-DHCR24-mRNA was applied to a carbon coated grid that was subjected to glow discharged for 1 min in air, and the grids were immediately negatively stained using 2% phosphotungstic acid for 60 s. Grids were examined in the TEM operated at 120 kV. Data are expressed as the mean ± SD. * *p* < 0.05 and ** *p* < 0.01 compared to the blank group.

#### 4.3.4. qPCR Detection

PCR reaction system: component 20 μL rxn 50 μL rxn; final concentration; 2×Fast SYBR Green qPCR Master Mix (High ROX): 10 μL; forward primer (10 μM): 0.4 μL; reverse primer (10 μM):0.4 μL; template: variable; nuclease-free water: added to 20 μL. Reaction system: 2× Fast SYBR Green qPCR Master Mix (High ROX) (10 μL), forward primer (10 μM, 0.4 μL), reverse primer (10 μM, 0.4 μL), nuclease-free water (added to 20 μL). The thermocycler program was as follows: 95 °C for 30 s and 40 cycles of 95 °C for 10 s, 60 °C for 30 s, and 72 °C for 20 s. The detection was performed on H9C2 rat cardiomyocytes, and the mRNA expression levels of DHCR24 in each group were compared. Data are expressed as the mean ± SD. * *p* < 0.05 and ** *p* < 0.01 compared to the blank group.

#### 4.3.5. Western Blot Detection

Total protein was extracted, BCA protein quantification (BCA Protein Concentration Determination Kit, Biosharp) was conducted, protein samples were denatured (1/4 of the sample volume was added to a 5× loading buffer, and they were mixed well, boiled and placed in a water bath at 95 °C for 5 min). SDS-PAGE electrophoresis and transmembrane were performed (according to the antibody instructions (1:1000)), and 6 μL of primary antibody was added. The mixture was incubated overnight at 4 °C on a shaker and then washed 3 times with 1× TBST for 10 min each time. The cleaned membrane was placed into the diluted secondary antibody (1:3000), incubated on a shaker at room temperature for 1 h and then cleaned three times with 1× TBST for 10 min each time, and ECL color development was observed. The protein expression levels of DHCR24 in each administration group of H9C2 rat cardiomyocytes and the protein expression levels of DHCR24 in each administration group of rat cardiac tissue were determined.

#### 4.3.6. Six-Minute Walk Test

A 50 cm × 50 cm square box made of transparent plastic or other materials that was easy to clean was prepared and observed. A timer was used to measure six minutes. Animals were ensured to have an appropriate adaptation period before testing. The animals were gently placed in the center of the square box. The timer was started, and the number of returns was recorded.

#### 4.3.7. ELISA Detection

The reagents were balanced at room temperature. The solutions were prepared, and the samples were added, incubated and washed. The enzyme-labeled reagents were added, incubated and washed. Color development was conducted. The stop solution was added, and the measurements were taken.

#### 4.3.8. Morphology and Pathology

After weighing the heart, it was fixed pathologically with 4% paraformaldehyde, embedded in paraffin and sectioned. HE staining was performed to observe the morphology and pathology of the cardiac tissue.

#### 4.3.9. B-Mode Ultrasonography

The rats were weighed and anesthetized. Their limbs were fixed and shaved. The coupling agent was applied, and a probe frequency of 10–15 MHz was used to evaluate the cardiac functions such as the ejection fraction (EF) of the rats.

GraphPad was utilized to conduct the analysis of the outcomes. Following a one-way ANOVA, Student’s *t*-test was applied to contrast the two conditions. The data are presented as the mean ± standard deviation. A *p*-value less than 0.05 was deemed to signify a statistically significant difference.

## Data Availability

Details are available from the authors.
